# Reverse Total Shoulder Arthroplasty for Proximal Humeral Fractures and Sequalae Compared to Non-Fracture Indications: A Matched Cohort Analysis of Outcome and Complications

**DOI:** 10.3390/jcm12062097

**Published:** 2023-03-07

**Authors:** Alexander Paszicsnyek, Philipp Kriechling, Sam Razaeian, Lukas Ernstbrunner, Karl Wieser, Paul Borbas

**Affiliations:** 1 Department of Orthopaedic Surgery and Traumatology, General Hospital Oberndorf, Paracelsusstraße 37, 5110 Oberndorf, Austria; 2 Department of Orthopedics, Balgrist University Hospital, 8008 Zürich, Switzerland; 3 Department of Orthopaedic Surgery, Royal Melbourne Hospital, 300 Grattan Street, Parkville, Melbourne, VIC 3050, Australia; 4 Department of Biomedical Engineering, University of Melbourne, Parkville, Melbourne, VIC 3010, Australia

**Keywords:** reverse total shoulder arthroplasty, proximal humeral fracture, complications, outcome, RTSA

## Abstract

Background: With the increase in utility and popularity of the reverse total shoulder arthroplasty (RTSA) within the last decades, indications for RTSA have expanded. As well as the established indications such as cuff tear arthropathy and massive irreparable rotator cuff tears, RTSA for complex proximal humeral fractures in elderly patients has been proven to be a reliable treatment option. Methods: A prospectively enrolled RTSA database of 1457 RTSAs implanted between September 2005 and November 2020 was reviewed. Patients treated with RTSA for a complex proximal humerus fracture and fracture sequalae (F-RTSA) were 1:1 matched with a group of patients who were treated electively with RTSA for indications other than a fracture (E-RTSA). Matching criteria included sex, age, length of follow-up and body mass index. Evaluation after a minimum of 2 years follow-up included evaluation of the absolute and relative Constant–Murley score (aCS; rCS), subjective shoulder value (SSV), range of motion (ROM) assessment and complications. Results: Each of the matched cohorts comprised 134 patients with a mean follow-up of 58 ± 41 months for the fracture group and 58 ± 36 months for the elective group. The mean age for both groups was 69 ± 11 years in the F-RTSA and 70 ± 9 years for the E-RTSA group. There were no significant differences in clinical outcome measures including aCS, rCS and SSV (*p* > 0.05). There was a significant difference in mean active external rotation with 20° ± 18° in the F-RTSA group compared with 25° ± 19° in the E-RTSA group (*p* = 0.017). The complication rate was not significantly different, with 41 complications in 36 shoulders in the F-RTSA and 40 complications in 32 shoulders in the E-RTSA group (*p* = 0.73). The main complication for the F-RTSA group was dislocation of the greater tuberosity (6%), whereas acromial fractures (9%) were the leading complication in the E-RTSA group. There was also no significant difference in revision rate comparing F-RTSA with E-RTSA (10% vs. 14%; *p* = 0.25). Conclusions: RTSA for complex proximal humeral fractures and its sequalae leads to a comparable clinical outcome as that for patients treated electively with RTSA for indications other than fracture. There was, however, a significant difference in active external rotation, with inferior rotation in patients undergoing RTSA for fracture. This valuable information can help in requesting informed consent of patients with proximal humeral fractures.

## 1. Introduction

Reverse total shoulder arthroplasty (RTSA) has continued to increase in utility and popularity since its introduction more than three decades ago [1]. During this period, improvements in the prosthetic design, surgical technique and surgeons’ experience have contributed to an expansion of indications for RTSA to treat a broad variety of shoulder disorders [1,2].

While initially indicated for patients with cuff tear arthropathy (CTA), RTSA indications include several other elective degenerative diagnoses such as massive irreparable rotator cuff tear with or without glenohumeral osteoarthritis (OA), rheumatoid arthritis, chronic dislocations, postinfectious sequelae, as well as tumor resection and the revision of failed anatomical or hemi-arthroplasties [2].

The addition of proximal humeral fractures (PHFx) as an indication for RTSA has led to an increased usage since the implant was approved by the US Food and Drug Administration in 2003 [3]. According to a review of a large sample of US Medicare data, the utilization of RTSA for the treatment of PHFx increased by over 400% between 2005 and 2012 [3,4].

Despite this rapid growth and the various number of degenerative disorders that RTSA is currently indicated for and FDA-approved to treat, there are only a few studies with inconsistent results that have investigated potential differences in outcomes between patients receiving RTSA for the treatment of PHFx and their sequelae versus RTSA indicated for degenerative conditions [1,3,5,6].

Performing RTSA for PHFx might not only present different surgical challenges to those for elective degenerative conditions but also encounter different patients’ demographics and expectations [1].

Precise knowledge about differences in outcomes following RTSA for various preoperative diagnoses might be clinically significant as it could assist surgeons in setting appropriate patient goals, improve informed consent and help to develop realistic expectations for patients’ recovery, as evidence suggests that patient outcomes might be related to their preoperative expectations [7,8,9].

Therefore, the aim of this matched cohort study was to investigate differences in clinical outcomes and complication rates between patients undergoing RTSA for elective degenerative conditions (E-RTSA) and those undergoing RTSA for the treatment of PHFx (F-RTSA). We hypothesized that clinical outcomes after PHFx are inferior compared to primary RTSA.

## 2. Materials and Methods

### 2.1. Patients

A prospectively enrolled RTSA database of 1457 RTSA implantations between September 2005 and November 2020 was reviewed. The F-RTSA group included patients with (1) acute fractures, (2) post-fracture OA, (3) Mal-/Non-Union and (4) humeral head necrosis after a PHFx. All included patients had a minimum clinical follow-up period of 2 years after RTSA implantation. The exclusion criteria were (1) a conversion or revision after anatomical total shoulder arthroplasty or hemiprosthesis.

The F-RTSA group was pair matched in a 1:1 manner to an E-RTSA group with regards to sex, age at surgery, side of surgery, length of follow-up and body mass index. The indication for RTSA in the E-RTSA group consisted of (1) cuff tear arthropathy, (2) primary OA and (3) irreparable rotator cuff tear.

### 2.2. Surgical Technique

All procedures were carried out by fourteen individual fellowship-trained shoulder and elbow surgeons. All procedures were performed using the Zimmer Reverse Anatomical Shoulder System (Zimmer, Warsaw, IN, USA) According to the indication, patients were treated with different RTSA stem designs. A fracture stem design was used in all acute fractures. In subacute fractures, a fracture stem was used only if the greater tuberosity had to be fixed to the stem as a separate fragment. This was performed with suture cerclages as previously described [10]. In the elective group a standard stem was used. The decision for cementation of the stem was made intraoperatively, depending on bone quality and the quality of press fit. All surgeries were performed through a deltopectoral approach by different senior shoulder surgeons as previously described [11,12]. The subscapularis tendon was detached in a peel-off fashion and reinserted with transosseous sutures at the end of the procedure. Postoperatively, all patients had the same standard follow-up treatment [13].

### 2.3. Outcome Measures

The following demographic data of all patients were collected: gender, side, age at surgery, BMI, ASA classification, smoking and alcohol consumption and previous surgeries. The indication for surgery was also documented for the F-RTSA and the E-RTSA group.

All patients were evaluated postoperatively at 6 weeks, 4.5 months, 1 and/or 2 years and then every 2–4 years. At each consultation, functional outcomes and conventional radiographic examination by an examiner different from the operating surgeon sequentially at each regular consultation and mean follow-up were determined. As outcome measurements, following scores were assessed: the absolute (aCS) [14] and relative Constant–Murley score (rCS) [15] including pain assessment [14,15], subjective shoulder value (SSV) [16,17] and range of motion (ROM) assessment. A subgroup analysis in the F-RTSA group was also carried out comparing outcomes for primary fracture of the proximal humerus to fracture sequalae (>6 weeks after initial trauma).

The definitions for minimal clinically important differences (MCIDs) are based on studies by Torrens et al. [18] and Simovitch et al. [19] The MCID cutoff for active flexion is 12°, abduction 7° and 3° for internal and external rotation. The anchor-based MCID for the absolute Constant score is 5.7 points.

The evaluation of complication was based on the above-mentioned database and sorted by complication and rate of revision. Radiographic notching and heterotopic ossification were not seen as complication due to no altering effect on outcome after RTSA [20,21].

### 2.4. Statistical Analysis and Data Collection

Study data were collected and edited using REDCap (Research Electronic Data Capture) tools [22,23]. Propensity score matching was performed using R Studio (MatchIt package; Vienna, Austria) for 1:1 matching using nearest pair function. All values are given as mean ± standard deviation or absolute number (percentage). Statistical analysis was conducted using students *t*-test for continuous data or chi-square test for categorial data. A *p*-value of < 0.05 was considered significant.

## 3. Results

### 3.1. Patient Demographics

For each fracture case, one control case was matched resulting in two groups of 134 patients each. Comparisons of age and gender were shown to be significantly different with a mean age of 69 ± 11 years in the F-RTSA and 70 ± 9 years for the E-RTSA group, and gender was dominantly female with 75% in the fracture and 77% in the E-RTSA group. The main indication for RTSA in the fracture group was a failure of the primary proximal humeral fracture treatment (failed osteosynthesis, posttraumatic osteoarthritis) with 63%, and for the E-RTSA group, irreparable rotator cuff tear with secondary OA (37%). Prior surgery was significantly more common in the elective group with an overall incidence of 48 for the fracture and 96 for the E-RTSA group (*p* < 0.01).

The mean follow-up was 58 ± 41 months for the F-RTSA and 58 ± 36 months for the E-RTSA group.

Detailed information on demographic data can be found in Table 1.

### 3.2. Range of Motion and Outcome Scores between F-RTSA and E-RTSA

Significant differences for outcome scores between both groups were only found regarding postoperative external rotation. The E-RTSA group was shown to provide better external rotation postoperative with 25° ± 19° of ER in comparison to 20° ± 18° in the fracture group (*p* = 0.017).

All other outcome measurements showed no significant differences between the two groups.

A detailed overview of all outcome scores can be found in Table 2.

### 3.3. Range of Motion and Outcome Scores in a Subgroup Analysis

No significant difference was found during the subgroup analysis of the fracture group. A general tendency towards superior outcome scores was seen for the acute fracture compared to the complications resulting from prior treatment of the fracture. The relative constant score and SSV was shown to be superior by 7% for the acute fracture group (rCS: 76 ± 20% vs. 69 ± 18%; *p* = 0.06; SSV: 75 ± 23 pts vs. 68 ± 24 pts; *p* = 0.1). The acute fracture subgroup also provided non-significant but borderline superior values for flexion (115° ± 33° vs. 106° ± 28°; *p* = 0.08).

A detailed summary can be found in Table 3.

### 3.4. Complications

An overall total of 40 complications in 32 shoulders (24%) was seen in the E-RTSA group, whereas 41 complications in 36 shoulders (27%) were seen in the F-RTSA group. There was no significant difference to be found between groups for the relative rate of complications (*p* = 0.73). The main complication in the E-RTSA group was acromial fracture (12/134, 9%) and dislocation of the greater tuberosity (8/134, 6%) in the F-RTSA group. Out of all shoulders, 19 shoulders (14.1%) in the E-RTSA and 14 shoulders (10.4%) in the F-RTSA group required revision. The main reason for revision was dislocation of the greater tuberosity (4/134, 3%) in the F-RTSA and acromial fracture (5/134, 4%) in the E-RTSA group.

Detailed data can be found in Table 4.

## 4. Discussion

This matched cohort analysis of patients undergoing treatment with reverse total shoulder arthroplasty in two different scenarios of indication (elective and PHFx) has provided new input for a hot topic of shoulder surgery with a paucity of literature.

The main finding of this study is that RTSA for proximal humeral fracture and its sequalae has—in contrast to the study hypothesis—a similar outcome in comparison to the primary RTSA without previous fracture. Although all outcome scores were slightly superior in the E-RTSA group, the F-RTSA group was shown to provide comparable postoperative results with no significant difference except for external rotation. The significantly increased ER for the E-RTSA group may be explained by the higher incidence of greater tuberosity fractures and therefore weakening of the external rotation in the fracture group. Although this was seen as a significant difference in our study, depending on the patient cohort, 5 degrees may be considered as clinically irrelevant.

In contrast to the present study, previous data found differences in the outcomes between patients undergoing RTSA for fracture or E-RTSA [5,24]. This was discussed as being caused by higher patient age, more dependent functional status and higher ASA scores in patients with PHFx previously [6]. Furthermore, a prolonged recovery as well as lower outcome scores were reported in a study by Paras et al. [5]. However, this meta-analysis might have a selection bias including there being more female patients of higher age and more frequent surgery on the non-dominant arm.

In order to avoid such a potential selection bias, we used a matched group study design with no differences in age and ASA score. A further possible reason for the similar outcomes in both groups in the present study is the increasing popularity with technical and fracture stem designs of RTSA for PHFx treatment and advances in tuberosity fixation techniques [25].

For this study, we also examined the difference in outcome and ROM within the F-RTSA group between acute and posttraumatic fracture RTSA in a subgroup analysis. Although we did not find a significant difference in outcome scores or ROM, the values provided show a tendency towards better outcomes for the acute fracture fixation using RTSA. This was also seen in the previous literature where the primary fracture RTSA was proven to be either better or comparable in outcome [26,27]. These aforementioned studies also reported a higher complication rate for the secondary RTSA [26,27,28], which was not investigated in this study. Therefore, RTSA as the primary treatment rather than delaying this procedure for osteosynthesis or the conservative treatment of complex proximal humeral fractures needs to be taken into consideration.

In our study, the fracture group implantation of a cemented stem was more common. This may lead back to the fact that cementing is used in the previously established technique for tuberosity fixation and healing. Said fixation may lead to improved fixation of the tuberosity but is known to result in inferior results and a higher incidence of adverse events in comparison to uncemented cases [29,30,31]. Although recent studies by Rossi et al. [32] and Joseph et al. [33] found the uncemented stem to be sufficient for fixation without an inferior outcome, cemented stem fixation is still common when RTSA implantation is chosen in a fracture case.

The effect of the minimal clinically important difference (MCID) may be seen as a reason for the non-significant differences in outcome scores and range of motion, with the values being defined as 12° for active flexion, 7° for abduction and 3° for internal and external rotation. The anchor-based MCID for the absolute Constant score is 5.7 points. Abduction differed by 6° between the E-RTSA and F-RTSA group in this study. This may be seen as clinically important, but due to the MCID we chose to include [18,19], it was not significantly different. In the previous literature, several values for the MCID in shoulder function were defined, resulting in a possible alteration of outcome significancy [34].

The outcome score was not significantly different between both study groups. In contrast to our findings, Paras et al. [5] found Constant scores for elective RTSA compared to fracture RTSA to be significantly improved for the elective indication. All other scores (ASES, SSV, RCS) were equal between groups corresponding to our study. The authors of the aforementioned study contributed the there being increased fracture involvement and a higher chance of adverse events affecting the greater tuberosity as one possible reason for worse outcome scores.

In addition to the patient specific factors, the technically more challenging procedure of RTSA in a traumatic or posttraumatic setting with altered anatomy could be another reason for inferior outcomes in the existing literature [5]. As mentioned previously, indications and techniques have advanced, which could explain the comparable results of F-RTSA in the present study.

Recent studies have shown that F-RTSA has a higher risk of adverse events. In a study published by Malik et al. [35], the proximal humerus fracture group was shown to provide a higher chance of revision within 30 days and a prolonged hospital stay. Therefore, patients undergoing elective RTSA may have a higher chance of a faster recovery with a lower complication rate. We did not investigate the early postoperative recovery in the present study. However, the F-RTSA group had a higher chance of adverse events by only 3%, which was not statistically significant.

Another interesting finding of our study is that the E-RTSA group had a high incidence of acromial fractures (9%). This fits well to the findings of Kriechling et al. [36], who published an overall rate of about 10% of acromial stress reactions including acromial stress fractures after primary RTSA. This may be caused by an earlier and faster recovery resulting in higher tension applied earlier on the deltoid in comparison to the F-RTSA group [5].

Additionally, the E-RTSA group had a slightly higher incidence of revision (14.1%) compared to RTSA (10.4%), despite there being no difference in the overall complication rate. This may lead back to the fact that the most common reasons for revision surgery in the E-RTSA group (material failure and acromial fracture) are associated with decreased patient satisfaction in comparison to the other included complications and can therefore lead to a revision more frequently [37,38].

We are aware that this study has several limitations. As well as its retrospective nature, our study consisted of different elective indications for surgery in comparison to the F-RTSA group. This led to heterogenous indications for the two study groups. Although we successfully matched our cohort through five key variables, this does not exclude a potential bias due to the heterogenous group to the F-RTSA group. Secondly, we did not investigate the postoperative therapy scheme as well as the patient compliance. Although our patients were treated in the same postoperative rehabilitation protocol, there may be still a potential source of difference resulting from the lack of compliance, resulting in differing results for rehabilitation. Lastly, this study was carried out by a single center and therefore only provided information on the specific RTSA model used in this institution.

## 5. Conclusions

RTSA for proximal humeral fractures and its sequalae leads to similar clinical outcome scores and complication rates as primary RTSA without previous fracture, with only significantly decreased external rotation function. This valuable knowledge can help surgeons seeking informed consent of patients with proximal humeral fractures.

## Figures and Tables

**Table 1:** Demographic data.

		**Fracture**	**Elective**	***p*** **-Value**
RTSA		134	134	
Gender	Male (%)	34 (25%)	31 (23%)	0.670
Side	Right side (%)	77 (57%)	73 (54%)	0.623
Age at Surgery		69 ± 11	70 ± 9	0.645
Body Mass Index		29 ± 6	29 ± 7	0.998
ASA Classification	ASA 1	10 (8%)	14 (10%)	0.484
	ASA 2	72 (54%)	72 (54%)	
	ASA 3	50 (37%)	48 (36%)	
	ASA 4	0 (0%)	0 (0%)	
	ASA 5	1 (1%)	0 (0%)	
	ASA 6	0 (0%)	0 (0%)	
Smoking (%)				0.342
	Never smoked	23 (17%)	18 (13%)	
	Stopped	20 (15%)	15 (11%)	
	Active	85 (63%)	98 (73%)	
	Unknown	6 (4%)	3 (2%)	
Alcohol (%)				0.176
	Unknown	14 (10%)	4 (3%)	
	No Alcohol	68 (51%)	74 (55%)	
	Rarely	39 (29%)	37 (28%)	
	Regularly	11 (8%)	16 (12%)	
	Abuse	2 (1%)	3 (2%)	
Cemented Stem (%)		86 (64%)	54 (40%)	<0.01
Previous Surgeries (%)		48 (36%)	95 (71%)	<0.01
	1	45 (34%)	23 (17%)	
	2	31 (23%)	9 (7%)	
	3	9 (7%)	5 (4%)	
	>4	1 (1%)	2 (1%)	
Indication				<0.01
	acute PHFx	49 (37%)		
	PHFx complication (>6 weeks)	85 (63%)		
	Primary OA		26 (19%)	
	Cuff tear arthropathy		14 (10%)	
	MIRCT		44 (33%)	
	MIRCT and OA		50 (37%)	

All values are given in mean ± standard deviation and absolute number (percentage of total). RTSA: reverse total shoulder arthroplasty; ASA: American Society of Anesthesiologists; PHFx: proximal humeral fracture; OA: osteoarthritis; MIRCT: massive irreparable rotator cuff tear.

**Table 2:** Outcome scores and range of motion.

		Fracture	Elective	*p*-Value
Number		134	134	
CSa	Preop	25 (15)	33 (15)	<0.01
	Postop	60 (17)	61 (20)	0.606
CSr (%)	Preop	32 (18)	41 (18)	<0.01
	Postop	72 (19)	73 (22)	0.573
SSV (%)	Preop	30 (21)	30 (19)	0.952
	Postop	71 (24)	74 (25)	0.268
CS Pain	Preop	7 (4)	6 (4)	0.044
	Postop	13 (3)	13 (3)	0.625
Flex (°)	Preop	59 (34)	84 (41)	<0.01
	Postop	109 (30)	112 (33)	0.519
Abd (°)	Preop	55 (30)	77 (38)	0.00
	Postop	114 (36)	120 (42)	0.177
ER (°)	Preop	11 (18)	25 (23)	0.00
	Postop	20 (18)	25 (19)	0.017
IR	Preop	3 (2)	4 (3)	0.00
	Postop	5 (3)	5 (3)	0.388
Force (Kg)	Preop	1 (1)	1 (2)	<0.01
	Postop	2 (2)	2 (2)	0.986
FUP (M)		58 (41)	58 (36)	0.961

All values are given in mean ± standard deviation. CSA: absolute constant score [14]; CSR: relative constant score [15]; SSV: subjective shoulder value [16,17]; CS: constant score [14]; Flex: flexion; Abd: abduction; ER: external rotation; IR: internal rotation; KG: kilograms; FUP: follow-up.

**Table 3:** Subgroup analysis acute proximal humeral fracture vs. post-acute proximal humeral fracture (>6 weeks post-trauma) of outcome scores and range of motion.

	Acute	Post-Acute	*p*-Value
RTSA (patients)	49 (48)	85(82)	
CSa	62 (17)	58 (17)	0.191
CSr (%)	76 (20)	69 (18)	0.056
SSV (%)	75 (23)	68 (24)	0.103
CS Pain	13 (3)	13 (3)	0.337
Flex (°)	115 (33)	106 (28)	0.079
Abd (°)	119 (36)	111 (35)	0.206
ER (°)	20 (20)	20 (16)	0.92
IR	5 (3)	5 (3)	0.143
Force (kg)	2 (2)	2 (2)	0.559
FUP (m)	50 (37)	63 (43)	0.075

All values are given in mean ± standard deviation and as absolute number (percentage of total). RTSA: reverse total shoulder arthroplasty; CSA: absolute constant score [14]; CSR: relative constant score [15]; SSV: subjective shoulder value [17,18]; CS: constant score [14]; Flex: flexion; Abd: abduction; ER: external rotation; IR: internal rotation; KG: kilograms; FUP: follow-up.

**Table 4:** Detailed description of complication and revision rate.

		Fracture	Elective
Shoulders (%)		36 (27%)	32 (24%)
Complications (%)		41	40
	Infection	8 (20%)	2 (5%)
	Nerve injury	7 (17%)	4 (10%)
	Material failure	5 (12%)	11 (28%)
	Periprosthetic fracture	8 (20%)	2 (5%)
	Greater tuberosity dislocation	9 (22%)	3 (8%)
	Acromial/scapular spine fracture	2 (5%)	12 (30%)
	Dislocation	2 (5%)	3 (8%)
	Persistent pain	0 (0%)	3 (8%)
Revision (%)		14 (10%)	19 (14%)
Reason for Revision			
	Infection	4 (29%)	1 (5%)
	Nerve injury	2 (14%)	5 (27%)
	Material failure	1 (7%)	1 (5%)
	Periprosthetic fracture	5 (36%)	
	Greater tuberosity dislocation		3 (16%)
	Acromial/scapular spine fracture		3 (16%)
	Dislocation		2 (11%)
	Persistent pain	2 (14%)	4 (21%)

All values are given as absolute number (percentage of total).
